# Incorporating statistical uncertainty in the use of physician cost profiles

**DOI:** 10.1186/1472-6963-10-57

**Published:** 2010-03-05

**Authors:** John L Adams, Elizabeth A McGlynn, J William Thomas, Ateev Mehrotra

**Affiliations:** 1 RAND, Santa Monica, CA, USA; 2 Muskie School of Public Service, University of Southern Maine, Portland, ME, USA; 3 Division of General Internal Medicine, School of Medicine, University of Pittsburgh, Pittsburgh, PA, USA; 4 RAND, 4570 Fifth Avenue, Suite 600, Pittsburgh, PA 15213-2665, USA

## Abstract

**Background:**

Physician cost profiles (also called efficiency or economic profiles) compare the costs of care provided by a physician to his or her peers. These profiles are increasingly being used as the basis for policy applications such as tiered physician networks. Tiers (low, average, high cost) are currently defined by health plans based on percentile cut-offs which do not account for statistical uncertainty. In this paper we compare the percentile cut-off method to another method, using statistical testing, for identifying high-cost or low-cost physicians.

**Methods:**

We created a claims dataset of 2004-2005 data from four Massachusetts health plans. We employed commercial software to create episodes of care and assigned responsibility for each episode to the physician with the highest proportion of professional costs. A physicians' cost profile was the ratio of the sum of observed costs divided by the sum of expected costs across all assigned episodes. We discuss a new method of measuring standard errors of physician cost profiles which can be used in statistical testing. We then assigned each physician to one of three cost categories (low, average, or high cost) using two methods, percentile cut-offs and a t-test (p-value ≤ 0.05), and assessed the level of disagreement between the two methods.

**Results:**

Across the 8689 physicians in our sample, 29.5% of physicians were assigned a different cost category when comparing the percentile cut-off method and the t-test. This level of disagreement varied across specialties (17.4% gastroenterology to 45.8% vascular surgery).

**Conclusions:**

Health plans and other payers should incorporate statistical uncertainty when they use physician cost-profiles to categorize physicians into low or high-cost tiers.

## Background

There is growing use of physician cost profiles which compare the costs incurred by a physician's patients to an expected level of costs [1-10]. These profiles, alone or in conjunction with quality profiles, are being used by health plans for public reporting or for the creation of selective or tiered networks. In a selective network, patients can only visit low-cost physicians. In a tiered network, patients pay a smaller co-payment to see low-cost physicians. For example Aetna's Aexcel network has two tiers of physicians and patients pay a $10-15 lower co-payment for visits with a low-cost, high-quality physician[5]. The goal of these initiatives is to create an incentive for physicians to decrease health care costs.

The physician cost profiles currently in use are based on commercial episode-grouper programs [2-9]. These programs group a patient's claims into different episodes of care which comprise all services (e.g., visits, laboratory tests, hospitalizations) for a patient for a specific condition. This condition-specific approach in theory better addresses patient case-mix differences between physicians[11]. However, there is the concern that despite this advantage, cost data are still inherently "noisy" and a physician could be labeled as high-cost simply because of the combination of statistical uncertainty ("noise") and insufficient sample size[1,5,12]. In statistical terminology this would be labeled Type 1 error.

To date health plans have generally not addressed statistical uncertainty when they identify physicians who are high, average, or low cost. Rather they use percentile cut-points across the distribution of physician cost profiles. For example, a health plan may label as low cost the 25% of physicians at the bottom of the distribution[5]. Based on one author's (JWT) consulting experience, health plans do not use statistical testing because of the lack of an available method and the concern that statistical testing will identify so few low-cost or high-cost physicians to make selective or tiered networks impractical. Under statistical testing many truly low-cost physicians might be labeled as average (Type II error in statistical terminology) because the p-value threshold typically used (0.05) is stringent.

In this paper we introduce a method for conducting statistical tests in the setting of physician cost profiles. We then compare the two methods, percentile cut-offs and statistical tests, on how many high or low-cost physicians they identify and whether they agree on assignment. Finally, we discuss the implications of our results on current policy and the concern that statistical testing identifies too few low or high-cost outliers.

## Methods

### Data sources and study population

We constructed an aggregated commercial claims data set that included all professional, inpatient, facility, and pharmacy claims from four health plans in Massachusetts for 2004-2005. We analyzed all claims for the 1.13 million enrollees between the ages of 18 and 65 who were continuously enrolled for the two years. We used a unique physician identifier previously created by Massachusetts Health Quality Partners to link data from the four health plans at the physician level[13]. Our study population consisted of Massachusetts physicians who submitted at least one claim to one or more of the four participating health plans and were in a non-pediatric, non-geriatric specialty with direct patient contact. Pediatricians and geriatricians were excluded because our claims sample was restricted to patients 18-65 years old. More details on our study population are provided in Additional file 1.

### Constructing physician cost profiles

Our methodology, which is described in greater detail in Additional File 1, was designed to replicate as closely as possible the cost profiling methods commonly used by health plans. It involved the following steps:

1. *Create standardized prices*. We averaged across the four health plans the mean allowed cost for each procedure, visit, service, or drug to create a standardized price for the state[14]. Before calculating the standardized prices we set all prices below the 2.5^th ^percentile to the price at the 2.5^th ^percentile and all prices above the 97.5^th ^percentile to the price at the 97.5^th ^percentile of the cost distribution, a process known as Winsorizing[15].

2. *Construct episodes of care*. We used Episode Treatment Groups^® ^(ETGs) software to aggregate each patient's claims into clinically related episodes of care (Version 6.0, Ingenix, Eden Prairie, Minnesota).

3. *Calculate each episode's observed costs*. The cost of each patient episode was calculated by summing the standardized costs of each service multiplied by the number of times the service was provided within the episode.

4. *Assign episodes to physicians*. The total cost of an episode of care was attributed to the physician who had billed the greatest fraction (minimum 30%) of professional costs within the episode.

5. *Calculate "expected" costs*. For each type of episode (e.g., uncomplicated diabetes) the expected cost was the mean cost across all episodes attributed to physicians of the same specialty, for patients with the same level of co-morbidities. We used Symmetry's Episode Risk Groups^® ^to assign patient episodes to different co-morbidity levels.

6. *Construct composite cost profile*. We calculated a ratio based on all episodes attributed to each physician:

### Creating standard errors for physician cost profiles

Statistical uncertainty of a physician's cost profile is measured by the standard error, which will differ from physician to physician depending upon the specific set of episodes assigned to that physician. Calculation of the standard error must account for the number of episodes assigned, the characteristics of each episode type (e.g. minor skin inflammation vs. hyperlipidemia), and the effects of patient comorbidity levels (risk-adjustment). Details are provided in Additional File 1, but in brief we define the variance of a physician's cost profile as:

This assumes that the variance of the sum of the expected costs is small compared to the sum of the observed costs because it is based on all the data.

For the Var(*Observed*) we can use the variance of the entire population for each particular ETG and comorbidity combination. The standard error is the square root of this variance.

### Two methods for categorizing physician performance

We compared two methods for putting physicians into the following three categories, low, average, and high cost. (We recognize that health plans sometimes only use two categories, average vs. low cost [5]. or average vs. high cost[16].) In the percentile cut-off method we rank ordered physicians based on their profile and labeled as low-cost and high-cost those in the bottom 25% and the top 25% respectively. We chose 25% as a cut-off as it is consistent with health plan initiatives[5,17]. In the second method we used a t-test to determine whether each physician's cost profile is significantly different from the mean cost profile within their specialty. The t-test was created by taking each physician's cost profile, subtracting the mean, and then dividing by the calculated standard error. In our primary analysis we used a p-value threshold of 0.05. In a secondary analysis we used a p-value of 0.20. We used a larger p-value to address the concern that statistical tests identify too few outliers (Type II error). As previously recommended by the National Committee for Quality Assurance we only profiled physicans with at least 30 assigned episodes[18].

We calculated how frequently the two methods disagree on the cost category assigned to each physician. We also measured disagreement using a weighted kappa. Under the weighted kappa method more weight is given to extreme disagreement (e.g., a physician labeled low cost under one method and high cost under the other) and less weight to less extreme disagreement (e.g., low cost under one method and average under the other).

All analyses were conducted in SAS version 9.1. (SAS Institute, Inc, Cary, NC).

## Results

Our analyses are based on a sample of 8689 physicians in 27 specialties who had 30 or more assigned episodes. In our database, there was significant variation among specialties in median number of episodes assigned (range 50 for psychiatry to 680 for dermatology) [Table 1]. Across the specialties, the standard deviation of episode costs assigned was much larger then the mean episode costs (e.g. among episodes assigned to cardiologists, $2,300 mean cost, $13,322 standard deviation) which is indicative of high variance.

**Table 1:** Number of episodes and costs of episodes assigned to different specialties

Specialty	Number of physicians	Median number of episodes assigned to physicians within specialty	Mean Costs per Episode ($)	Standard Deviation of Costs per Episode ($)
All Specialties	8689			

Allergy and Immunology	77	177	795	2936

Cardiology	474	92.5	2300	13322

Cardiothoracic Surgery	27	54	13488	34751

Dermatology	311	680	260	685

Emergency Medicine	521	97	768	2493

Endocrinology	97	116	975	2601

Family/General Practice	848	418.5	310	1447

Gastroenterology	356	292.5	1114	2683

General Surgery	407	142	2605	18993

Hematology/Oncology	122	93.5	2838	13420

Infectious Diseases	89	165	492	2160

Internal Medicine	2158	392.5	379	2317

Nephrology	111	67	1085	5131

Neurological Surgery	62	55	6936	18639

Neurology	249	108	1297	4605

Obstetrics and Gynecology	756	346	958	3554

Ophthalmology	421	326	358	913

Oral & Maxillofacial Surgery	113	67	674	2097

Orthopedic Surgery	451	140	2008	9667

Otolaryngology	191	302	698	7164

Physical Medicine & Rehab	70	74	1453	4586

Plastic Surgery	101	119	1150	3869

Psychiatry	97	50	2320	3904

Pulmonary & Critical Care	203	120	856	5555

Rheumatology	127	190	958	3958

Urology	191	242	1473	5547

Vascular Surgery	59	100	3223	14119

Based on the t-test with a p-value of 0.05, 25.2% of all physicians were placed in either the high- or low-cost category (range across specialties 5.1-66.2%) [Table 2]. Using a p-value of 0.20, 42.1% of all physicians were placed in either the high- or low-cost category (range across specialties 14.8% to 75.9%).

**Table 2:** By specialty comparison of two methods for categorization and percentage of physicians identified as high cost or low cost

			T Test (p = 0.05)				T Test (p = 0.20)			
**Specialty**	**Number of physicians (≥ 30 episodes)**	**Physicians assigned a different cost category when comparing t-test (p = 0.05) and percentile cut-off (%)**	**Fraction of physicians identified as high cost using t-test N (%)**		**Fraction of physicians identified as low cost using t-test N (%)**		**Fraction of physicians identified as high cost using t-test N (%)**		**Fraction of physicians identified as low cost using t-test N (%)**	

All Specialties	8689	29.5%	1072	12.3%	1120	12.9%	1652	19.0%	2004	23.1%

Allergy and Immunology	77	27.3%	20	26.0%	19	24.7%	22	28.6%	30	39.0%

Cardiology	474	34.0%	51	10.8%	34	7.2%	65	13.7%	99	20.9%

Cardiothoracic Surgery	27	44.4%	2	7.4%	0	0.0%	3	11.1%	1	3.7%

Dermatology	311	21.2%	102	32.8%	104	33.4%	117	37.6%	119	38.3%

Emergency Medicine	521	34.9%	43	8.3%	37	7.1%	85	16.3%	70	13.4%

Endocrinology	97	33.0%	10	10.3%	10	10.3%	21	21.6%	15	15.5%

Family/General Practice	848	28.4%	102	12.0%	103	12.1%	173	20.4%	199	23.5%

Gastroenterology	356	17.4%	77	21.6%	59	16.6%	101	28.4%	88	24.7%

General Surgery	407	39.6%	23	5.7%	22	5.4%	39	9.6%	53	13.0%

Hematology/Oncology	122	36.1%	11	9.0%	7	5.7%	24	19.7%	26	21.3%

Infectious Diseases	89	32.6%	8	9.0%	13	14.6%	12	13.5%	18	20.2%

Internal Medicine	2158	24.6%	292	13.5%	382	17.7%	440	20.4%	625	29.0%

Nephrology	111	40.5%	6	5.4%	7	6.3%	14	12.6%	16	14.4%

Neurological Surgery	62	33.9%	7	11.3%	4	6.5%	11	17.7%	10	16.1%

Neurology	249	24.1%	35	14.1%	31	12.4%	56	22.5%	50	20.1%

Obstetrics and Gynecology	756	36.4%	54	7.1%	55	7.3%	91	12.0%	130	17.2%

Ophthalmology	421	18.3%	86	20.4%	89	21.1%	113	26.8%	128	30.4%

Oral & Maxillofacial Surgery	113	32.7%	14	12.4%	7	6.2%	24	21.2%	18	15.9%

Orthopedic Surgery	451	39.7%	20	4.4%	29	6.4%	58	12.9%	76	16.9%

Otolaryngology	191	19.4%	30	15.7%	37	19.4%	46	24.1%	66	34.6%

Physical Medicine & Rehabilitation	70	27.1%	8	11.4%	9	12.9%	12	17.1%	16	22.9%

Plastic Surgery	101	34.7%	10	9.9%	7	6.9%	20	19.8%	19	18.8%

Psychiatry	97	30.9%	7	7.2%	13	13.4%	9	9.3%	25	25.8%

Pulmonary & Critical Care	203	40.9%	15	7.4%	8	3.9%	27	13.3%	25	12.3%

Rheumatology	127	29.1%	17	13.4%	16	12.6%	24	18.9%	37	29.1%

Urology	191	31.9%	20	10.5%	17	8.9%	37	19.4%	43	22.5%

Vascular Surgery	59	45.8%	2	3.4%	1	1.7%	8	13.6%	2	3.4%

When comparing percentile cut-offs and t-tests (p-value ≤ 0.05), the two methods assigned 29.5% of physicians to different cost categories [Table 3]. The weighted kappa was 0.53 which would be interpreted as "moderate" agreement using the classification proposed by Landis[19]. Levels of disagreement varied across specialties, ranging from 17.4% for gastroenterology to 45.8% for vascular surgery. In supplementary analyses described in Additional File 1 we calculated the level of disagreement of t-tests and percentile cut-offs using an analysis where the fraction of high-cost and low-cost physicians is fixed across the two methods. In this supplementary analysis there was also substantial disagreement.

**Table 3:** Comparison of two methods for categorizing physicians

Cut-off method	T-test method with p-value ≤ 0.05			Total
	**High Cost** **n(%)**	**Average Cost** **n(%)**	**Low Cost** **n(%)**	

**High Cost**	1030 (11.9)	1154 (13.3)	0 (0.0)	2184 (25.1)

**Average Cost**	42 (0.5)	4127 (47.5)	152 (1.8)	4321 (49.7)

**Low Cost**	0 (0.0)	1216 (14.0)	968 (11.1)	2184 (25.1)

**Total**	1072 (12.3)	6497 (74.8)	1120 (12.9)	8689 (100.0)

## Discussion

There is growing use of physician cost profiles in an effort to decrease health care costs. Most of the policy applications of physician cost profiles such as selective or tiered networks require that physicians are divided into categories. Our results suggest that the method currently used by health plans to create these categories, percentile cut-offs, is inappropriate because it does not account for the substantial statistical uncertainty inherent in physician cost profiles. We demonstrate that when comparing the two methods, percentile cut-offs vs. statistical testing, almost a third of physicians are put into a different cost category. We advocate for the use of statistical testing in future cost profiling applications.

As noted above, health plans have been wary of using statistical testing because of concern that too few physicians will be placed into the low-cost or high-cost tiers and therefore making tiered networks impractical. In our study approximately 25% of physicians were put into the low-cost or high-cost category based on a t-test with a p-value cut-off of 0.05. Whether this is too many or too few depends on one's perspective. Using a p-value of 0.05, minimizes Type 1 error (e.g., physicians who are *not *high-cost are misclassified as high-cost), but likely results in significant Type 2 error (e.g., physicians who are high-cost are misclassified as average). Physicians would prefer to minimize Type 1 error in this situation because being labeled as high-cost may damage their reputation as well as hurt them financially. Health plans are more likely to be concerned with Type 2 error. They hope cost profiles force physicians to focus on inappropriate spending. If a large number of high-cost physicians are misclassified as average, then the impact of the profiling efforts will be diminished.

There are alternative choices for statistical testing that might provide a better balance between these different viewpoints and their concern about the two types of statistical error. We illustrated one alternative using a p-value of 0.20 which likely increases Type 1 error and decreases Type 2 error. Under this alternative method, the number of outliers in our data increased from 25% to 42%. This might be a sufficient number of outliers from the perspective of a health plan which is trying to create a tiered network. A second alternative, initially proposed by one of our co-authors, [20] is to rank order t-test values of physician efficiency measures and use a percentile cut-off (e.g. 20%) of this t-test distribution. This method has the advantage that a fixed percentage of outliers are identified for each specialty. The disadvantage is that for some specialties a high p-value threshold will be necessary to identify this fixed percentage of outliers. A high p-value threshold greatly increases the Type 1 error (e.g. physicians who are *not *high-cost are misclassified as high-cost). This method is described in more detail in Additional File 1. A third alternative is to reframe the statistical testing question. For example, the t-test could be reframed as the probability that the physician is different than the average physician in the lowest-quartile (as opposed to the average physician overall). These alternative approaches demonstrate that there are mechanisms to increase the number of outlier physicians while still addressing statistical uncertainty.

There are several important limitations to this research. We used a parametric approach to estimate the standard errors for the physician cost profiles. A non-parametric approach, for example using a null bootstrap estimator, might be more accurate. We did not use a bootstrap approach, because running bootstraps is often less intuitive for a policy and health plan audience and requires intensive computer resources. We felt a parametric approach would be more likely to be used in practice. Our analyses are based on data from four Massachusetts health plans. Although the specific level of disagreement will be different in other settings, we believe our overall finding of substantial disagreement is likely generalizable. Lastly, this research does not address many other methodological issues that need to be addressed in developing cost profiles including the validity of the episode-grouper programs themselves and how care is assigned to a physician.

## Conclusions

Using cost profiles to place physicians into high, average, or low-cost categories is becoming more common. We advocate that this categorization should address the statistical uncertainty inherent in profiles and we introduce a method of doing so.

## Competing interests

JWT has received consulting support on the topic of physician cost profiling from Agency for Healthcare Research and Quality, American Board of Medical Specialties, American Medical Association, Arkansas Medical Association, Blue Cross Blue Shield of Michigan, CIGNA Healthcare, Integrated Healthcare Association, Massachusetts Medical Society, Pacific Business Group on Health, Wisconsin Collaborative for Healthcare Quality, and the Wisconsin Medical Association. The authors have received a grant from the Massachusetts Medical Society, American Medical Association, and Physicians Advocacy Institute to study other aspects of physician cost profiling. None of the authors have any other financial interest in or a financial conflict with the subject matter or materials discussed in this manuscript.

## Authors' contributions

JLA conceived of the idea and provided statistical oversight. JLA, EAM, JWT, AM participated in its design of the study and writing of the manuscript. EAM conceived of the larger project and obtained funding. AM oversaw the analyses and wrote the initial draft of the manuscript. All authors read and approved the final manuscript.

## Pre-publication history

The pre-publication history for this paper can be accessed here:

http://www.biomedcentral.com/1472-6963/10/57/prepub

## Supplementary Material

Additional file 1 **Technical appendix to accompany paper entitled "Incorporating statistical uncertainty in the use of physician cost profiles"**. The purpose of this technical appendix is to provide more detail about the methods in the manuscript in particular on how we calculated the standard errors of physician cost profiles as well as supplemental analyses on how we compare the two physician categorization systems. Click here for file
