# Supplementary material for: Incorporating statistical uncertainty in the use of physician cost profiles
Source: BMC Health Serv Res. 2010 Mar 5;10:57. doi: 10.1186/1472-6963-10-57 (PMC2842268; doi:10.1186/1472-6963-10-57)
Supplement: Additional file 1 — Technical appendix to accompany paper entitled "Incorporating statistical uncertainty in the use of physician cost profiles". The purpose of this technical appendix is to provide more detail about the methods in the manuscript in particular on how we calculated the standard errors of physician cost profiles as well as supplemental analyses on how we compare the two physician categorization systems. [file 1472-6963-10-57-S1.DOC]

**Technical appendix to accompany paper entitled “Incorporating statistical uncertainty in the use of physician cost profiles”**

1. **INTRODUCTION**

This purpose of this technical appendix is to provide more detail about the methods in the manuscript in particular on how we calculated the standard errors of physician cost profiles as well as supplemental analyses on how we compare the two physician categorization systems. Some material that appears in the manuscript is also reported here so that the technical appendix is a self-contained document.

The technical appendix is organized as follows:

- Section II provides details on the data sources and inclusion criteria for patients and physicians in the study;
- Section III describes the methods used in this study to construct physician cost profiles;
- Section IV provides details about how we calculated the standard errors for the physician cost profiles
- Section V describes the supplemental analyses where we compare the two physician categorization systems controlling for the fraction of outliers

1. **DATA SOURCES AND CRITERIA FOR INCLUSION OF PATIENTS AND PHYSICIANS**

**A. Source of Claims Data**

We obtained all commercial professional, inpatient, other facility, and pharmaceutical claims in 2004-2005 from four health plans in Massachusetts. Because most physicians contract with multiple health plans, we aggregated data at the physician level across health plans to construct a larger number of observations on each physician than would be possible for a single health plan. The dataset includes claims from managed care, preferred provider organization (PPO), and indemnity product lines. At the time of the study, the four health plans enrolled more than 80% of residents with any type of commerical health insurance in Massachusetts.

**B. Selecting Patients for Inclusion in the Study Population**

Our analyses used all claims for adult enrollees between the ages of 18 and 65 who were continuously enrolled for the two year period (2004-5) and who filed at least one claim. Figure A.1 shows the steps we took to arrive at the final study sample, and the implications of those steps for the final sample size.

We excluded patients over the age of 65, because these patients are eligible for Medicare and the plans could not reliably identify those for whom Medicare was the primary payer. Exclusions of children (<18) and those who were not continuously enrolled were necessary for another aspect of the project that examines the relationship between cost and quality performance. As a result, we included 58% of non-elderly adults in the study.

**Figure A.1: Steps in Selecting Patients for Inclusion in Study**

**C. Physician Database and Selecting Physicians for Inclusion in the Study**

Massachusetts Health Quality Partners (MHQP) created a master physician database for the state for the purposes of aggregating data across health plans and for public reporting of quality information. This database includes all physicians who are listed on the provider files of the largest health plans in Massachusetts. For each physician, MHQP creates a master physician ID and links that to the physician identifier used by each of the health plans. MHQP matches physicians across the health plans using unique identifiers (e.g., Massachusetts license number), names, and addresses. MHQP also determines physician specialty using the specialty listed in the health plan provider files. In the final reconciliation, MHQP used the Massachusetts Board of Registration file to verify mismatched license numbers and clinical specialties.

In the master physician database created by MHQP, approximately 20% of physicians had two specialties listed. For this project we assigned each physician to a single specialty using the following logic:

1. In most cases, the combinations were a general specialty (e.g., internal medicine) and a related subspecialty (e.g., cardiology). Because subspecialty costs are typically higher and the main use of specialty in our analyses was to compare each physician’s cost profile to the average of other physicians in the same specialty, we used the subspecialty because that would decrease the likelihood that a physician would be identified as a high cost outlier.
2. If one of the two specialties was a non-direct patient care specialty (e.g., pathology and internal medicine) then we selected the direct patient care specialty (internal medicine).
3. In the very rare cases where there was no clear hierarchy between the two specialties (general surgery vs. pulmonary & critical care) then we selected the first listed specialty (the order had been previously assigned at random by MHQP).

The use of this master physician database allowed us to link a physician’s claims across health plans. There are 30,004 physicians listed in this database. From this list of 30,004 we first restricted our sample to the 21,077 physicians with a Massachusetts address who had filed at least one claim with any of the four health plans in 2004-2005 (see Figure A.2 below). We excluded physicians who have retired, practice outside the state, left the state, or those with temporary licenses such as residents.

We then excluded the following physicians: (1) pediatricians and geriatricians, to be consistent with our patient exclusion rules; (2) those in non-direct patient care specialties (e.g., anesthesiologists, pathologists, radiologists; (3) those without a specialty assigned; (4) non-physicians (e.g., chiropractors, clinical psychologists); and (5) those in specialties with fewer than 75 total members in the state [to allow an adequate sample for constructing peer group comparisons]; (6) those who were not assigned any episodes (7) as recommended by the National Committee for Quality Assurance those with less than 30 assigned episodes.(1) The final sample contained 8,689 physicians (see Figure A.2).

**Figure A.2: Defining Physician Sample**

**III. CONSTRUCTING COST PROFILES**

We describe here the steps we used to construct physician-level cost profiles. For the purposes of this study, we made choices that reflect what health plans commonly do in constructing these profiles. These should be viewed as stylized or prototypical cost profiles. It was outside of the scope of our project to design an optimal method of physician cost profiling, rather we set out to understand the implications of some of the methods that were in common use.

The basic steps involve: (1) selecting a method for aggregating claims into meaningful clinical groupings to facilitate case-mix adjustment, called episodes in our analysis; (2) developing a method for attaching prices to episodes; (3) attributing episodes to physicians; (4) constructing a composite cost profile for each physician. Each step is described below in more detail than space would allow in the paper.

**A. Creating Episodes of Care**

We used Ingenix’s Episode Treatment Groups® (ETG) program, Version 6.0, which is a commercial product commonly used by health plans, to aggregate claims into episodes. We chose this commercial program because was being used by the health plans that were participating in our study and the version because that was the one for which we had a research license for this study.

The ETG methodology is described in detail in previous publications.(2) Briefly, the software evaluates all types of claims (inpatient, outpatient, and ancillary) and sorts them into mutually exclusive and exhaustive episode types or ETGs. There are 574 different episode types; examples include “hypo-functioning thyroid gland”, “viral meningitis”, and “cataract with surgery”.

The duration of an episode is flexible. For an acute illness the start and end of an episode is defined by a “clean-period”, a pre-specified time period before and after the claim that identified or “triggered” the episode during which no treatment occurs (often 30 days). For chronic illness episodes no clean period is required and the duration is generally one year. A patient can have concurrent episodes that account for different illnesses occurring at the same time. For example, a chest x-ray and blood glucose exam performed during the same encounter may be assigned to separate episodes if the patient is treated simultaneously for both bronchitis and diabetes.

As is standard we used only complete episodes or, for chronic illnesses, episodes that spanned a full year (designated by ETG types 0, 1, 2, or 3). Except for our method for addressing outliers and assigning costs to each service, we used the default settings for the program (i.e., we used the default clean periods).

**B. Attaching Prices to Episodes**

In attaching prices to episodes, we had to choose between using the actual prices paid by health plans and creating a standardized price across all plans. Conceptually using standardized costs is using the standardized values to weight utilization in the same way for all physicians. Since physicians do not normally negotiate their own prices this focuses the profiling on their actions rather than on differences in contractual features. We describe here the method for creating standardized prices.

In creating the standardized prices, we used the “allowed cost” field from the claims, which captures the total amount that can be reimbursed for a service including co-payments. We summed the allowed costs for each type of service (procedure, visit, service, drug) across health plans and divided by the number of services of that type to arrive at an average price.

Although each health plan paid for inpatient admissions based on DRGs, the four plans used different and incompatible systems so the standardized price applied to a hospitalization was the average lump sum reimbursement for that DRG *within a given* health plan. Only facility reimbursements were included. A small fraction of hospitalizations were assigned more than one DRG. These hospitalizations were not included in the calculations of standardized prices and the standardized price we applied to these hospitalizations was for the DRG with the highest average price.

To account for likely data errors, we set all allowed costs below the 2.5th and above the 97.5th percentile of each service cost distribution to the values at those cutpoints, a process known as Winsorizing. We selected Winsorizing over other methods of dealing with outliers because our prior work found that was a superior method.(3)

We then created a standardized cost for an episode by multiplying the number of units of each service delivered by the standardized price for that service. We Winsorized the standardized cost for each episode by setting total costs falling below the 2.5th and above the 97.5th percentile distribution to the values at those cutpoints. We refer to the resulting total cost of an episode as the observed cost.

**C. Attributing Episodes to Physicians**

Because in most situations no accountability is assigned a priori to a physician for a patient’s care, algorithms have been developed to make such assignments based on different patterns of utilization. These algorithms are broadly referred to as attribution rules.

In other work on this project we have evaluated 12 different attribution rules that use different combinations of the unit of analysis (episode or patient), signal for responsibility (visits or costs), thresholds for assigning responsibility (plurality or majority), and number of physicians to whom care is attributed (single or multiple). For this paper, we report results using the attribution rule that appears to be most commonly used by health plans, responsibility for episodes is assigned to the physician who bills the highest proportion of professional costs (defined below) in an episode as long as that proportion is greater than 30%. If no physician met the criteria, the episode was dropped from our analyses. A total of 48% of all episodes could *not* be assigned to a physician.

a. Defining Professional Costs for the Purposes of Attribution

There is no standard definition of what is a professional cost or claim. Typically professional services exclude facility and direct medical equipment.   Broader definitions might include ordering of pharmaceuticals, diagnostic imaging, laboratory tests, or pathology specimens while stricter definitions might exclude these services. For the purposes of this project we utilized a stricter definition because it is often difficult to determine which provider ordered a laboratory test, imaging test, or prescription because ordering physician is inconsistently recorded in health plan claims data.

Our goal was to use codes in which the delivering physician played a role in evaluating the patient or choosing a therapy. We are cognizant that no definition is perfect, and there may be disagreements on specific scenarios.

For our definition we started with all procedures on the 2007 Medicare National Physician Fee Schedule Relative Value File which includes all services (defined via CPT/HCPCS codes) rendered by providers and their subsequent RVU rates for Medicare. We then took the subset of codes in the following relevant Berenson-Eggers type of service code (BETOS) categories(4) [BETOS is a system developed by the Centers for Medicare and Medicaid Services to group care into more clinically relevant categories]: (1) Evaluation & Management except M5A [Specialist pathology] (2) Procedures except P0 [Anesthesia], (3) I4A Imaging/procedure except 0152T, (4) the following I4B imaging/procedure codes (0024T, 0062T, 0063T, 36100, 36200, 36160, 36010, 72291, 72292, 75901, 75902, 75958,75959, 76000, 76001, 75956, 75957), (5) Other (includes chiropractic care, delivery of medications, immunizations, vaccines) except O1A [Ambulance], and (6) Unclassified (Y) (includes items such as shoulder surgery, physician standby services, birth attendance, certain medication delivery). In selecting these 6 categories we would thereby eliminate most imaging, tests, durable medical equipment, and Z codes (exceptions, local, undefined codes).

**D. Calculate expected (average) costs**

The observed costs for a given episode were compared to what is “expected”. Expected was calculated by taking the average cost among episodes among patients assigned to physicians within the same specialty with the same level of co-morbidities and severity of illness (how this is measured is described below). We chose to compare costs within specialty because it is the method most frequently used by health plans and is seen another mechanism to control for severity of illness. It is likely that a hypertension episode assigned to a primary care physician is different than a hypertension episode assigned to a nephrologist.

a. Adjusting for co-morbidities

The Episode Risk Groups® classification system created by Symmetry assigns a patient’s episode to a discrete risk level based on a risk-adjustment methodology that is based on current and future health care costs as well as demographic variables. The number of risk levels varies by episode type. Some episodes have 4 levels and others have only 1 level. This depends on the relationship between co-morbidities and costs observed by Symmetry in their test database. The risk level for a patient is also not uniform. For example, a patient could be assigned the highest cost risk level for diabetes, but be assigned a middle risk level for hypertension. We used the default settings for the Episode Risk Groups® classification.

**E. Constructing a Composite Cost Profile Score**

We calculated the composite cost profile score as a ratio based on all episodes attributed to each physician:

Sum of the Observed Costs

Composite Cost Profile = ---------------------------------

Sum of the Expected Costs

Or in mathematical notation:

$$O/E=(\sum Observed_{i})/(\sum Expected_{i})$$

Where the sum (i) of the observed is over all of the episodes attributed to the physician and the sum (i) of the expected is the sum of the averages of the equivalent set of episodes attributed to all physicians in the same specialty

If the sum of observed costs exceeds the sum of expected or average costs (i.e., the physician is more costly than his/her peers), the physician’s cost profile score will be greater than one. If the sum of observed costs is lower than the expected or average costs (i.e., the physician is less costly than his/her peers), the cost profile score will be less than one. The composite cost profile is a continuous variable with a median near one, a minimum value of near zero, and no bound on the maximum value. Very low (close to zero) observations are seldom observed in our data and the maximum value rarely exceeds 10. The use of division for the ETG adjustment is analogous to the standardized mortality ratio or other adjusted metrics designed to maintain a mean near one and a proportion or percentage interpretation. It is perhaps surprising that the distributions are reasonably symmetric. The mean is rarely more than 20% higher than the median. Most cost distributions are skewed even after case mix adjustment. The symmetry is a consequence of the O/E metric and the detailed adjustment of the ETGs.

**IV. Calculating the standard errors of physician scores**

The ratio of observed to expected costs (O/E) is the physician efficiency metric used in the paper. The definition is:

$$O/E=(\sum Observed_{i})/(\sum Expected_{i})$$

where the sums are over all of the episodes attributed to a physician. A particular type of episode may appear multiple times in each sum if several episodes of that type are attributed to the physician. One point of clarification, when we refer to episode type, we go to the most disaggregated refinement of the ETG system. Specifically this is the ETG by ETG sub by ERG severity class cell. ETG subs are situations where a given ETG (e.g. bone fracture) is divided into sub-levels based on location of fracture (e.g. leg vs. hip) or type of treatment (e.g. surgery vs. no surgery).

The variance of a physician’s score can be derived with a few simplifying assumptions:

$$Var(O/E)=\mathrm{var}((\sum Observed_{i})/(\sum Expected_{i}))$$

$$Var(O/E)=\mathrm{var}(\sum Observed_{i})(\sum Expected_{i})^{-2}$$

assuming the variance of the expected values is small compared to the variance of the similar components of the physician’s score. This will be true if there are enough physicians that the expected values are based on much larger sample sizes than an individual provider’s score. Assumptions of this type are common in risk adjustment system standard error calculations. This assumption could be relaxed in smaller data sets via a delta method calculation to incorporate the variance of the expected values. We chose not to pursue the delta method calculation as it led to only a modest increase in variance from 0.5% for internal medicine to 1.2% for family practice. This difference does not seem to be worth the added complexity of using the delta method.

$$Var(O/E)=(\sum\mathrm{var}(Observed_{i}))(\sum Expected_{i})^{-2}$$

This theoretical formula needs to be implemented by plugging in estimates of the theoretical quantities. For the expected values we plug in the means of the corresponding episode type. The$\mathrm{var}(Observed_{i})$is a more problematic quantity to estimate, especially for the rare episode types in some specialties. The first choice that must be made is whether the logic of the standard error should focus on statistical testing or confidence interval construction. In this paper we can focus on testing. This allows us to calculate the standard errors under the typical null hypothesis assumption that the physician’s costs are a random sample from the population. Since cost data is often log-normal, different assumptions would be used if the goal were confidence intervals rather than tests. Under the null hypothesis we can plug in the variance of the entire specialty’s population for each particular episode type. The standard error is the square root of this variance.

This strategy breaks down for episode types that only have a single observation within a specialty. This occurs for 4123 episodes (of the over 3 million in our analytic sample). In this case we assume a coefficient of variation of one and impute the variance from the mean for the cell. Within the ETG system this coefficient of variation assumption is well supported by the empirical relationships in the ETG by ETG sub by ERG severity class cells where we can estimate a variance. As an example figure A.3 shows the standard deviation to mean relationship for the ETG by ETG sub by ERG severity class cells in log scale for one specialty.

**Figure A.3: Relationship Between Mean Cost of Episodes and Standard Deviation of Costs Across Episode Types**

SD of Episode Costs

Log of mean cost of episodes

**V. Comparing assigning physicians to categories with percentile cut points vs. statistical tests.**

In the manuscript we compare the categorization of physicians into cost bins using two techniques, 1) percentile cut-offs and 2) statistical testing. Overall we find that 29.5% of physicians are put in a different cost category. The comparison for internal medicine in demonstrated in Table A.1 below. If we look at the cells “off-diagonal” their sum is 24.6% (10.2 + 0.1 + 11.5 + 2.8). This is the fraction of internal medicine physicians are put in a different cost category when we compare statistical testing with a p-value cutoff of 5% to a cut point method using 25% thresholds on both ends of the distribution.

**Table A.1**. – Comparison of Two Techniques in Categorizing Internal Medicine Physicians

|  |  | Using t-test method p≤0.05 | | |  |
| --- | --- | --- | --- | --- | --- |
|  |  | Low-cost | Average | High Cost | Total |
| Using cutpoint  method | Low-cost | 14.9 | 10.2 | 0.0 | 25.0 |
|  | Average | 2.8 | 47.1 | 0.1 | 50.0 |
|  | High-Cost | 0.0 | 11.5 | 13.5 | 25.0 |
|  | Total | 17.7 | 68.8 | 13.5 | 100.0 |

In making this comparison we chose a commonly use percentile cut-off technique and the most classic form of statistical testing; we test whether a physician is different than the mean physician in their specialty based on a p-value of 0.05.

But we recognize one concern with such a comparison is that under the two categorization methods there are a different fraction of outliers. Table A.2 shows the fraction of physicians who are outliers under the two techniques. The difference in percentage of outliers “forces” some degree of disagreement.

**Table A.2**– Fraction of Physicians Categorized as Either High or Low Cost Outliers

|  | Internal Medicine |
| --- | --- |
| Using cutpoint method | 50% |
| Using t-test method p≤0.05 | 24.6% |

One way to “control” for the difference in fraction of physicians labeled as outliers is to decompose the 3x3 table shown above into the following 5x5 table:

**Table A.3 – Comparison of Two Techniques in Categorizing Internal Medicine Physicia**ns

| **Using cutpoint method** | **Using t-test method** | | | | | **Total** |
| --- | --- | --- | --- | --- | --- | --- |
|  | **low cost (p≤0.05)**  **0-**  **17.7%** | **17.7- 25%** | **25% to 75%** | **75% to 86.5%** | **High cost (p≤0.05)**  **86.5-100%** |  |
|  |  |  |  |  |  |  |
| **0-**  **17.7%** | 11.4 | 2.2 | 4.2 | 0.0 | 0.0 | 17.8 |
| **17.8- 25%** | 3.5 | 1.3 | 2.4 | 0.0 | 0.0 | 7.3 |
| **25% to 75%** | 2.8 | 3.8 | 39.6 | 3.7 | 0.1 | 50.0 |
| **76% to 86.5% (75 + 11.5%)** | 0.0 | 0.0 | 3.3 | 4.8 | 3.3 | 11.5 |
| **86.5- 100%** | 0.0 | 0.0 | 0.5 | 3.0 | 10.2 | 13.6 |
| **Total** | 17.7 | 7.3 | 50.0 | 11.5 | 13.5 | 100.0 |

In this 5x5 table we have added two percentile cut-off rows:

1. Percentile cut-off of 17.8% because 17.8% of the internal medicine physicians were statistically different low-cost outliers.
2. Percentile cut-off of 86.5% because 13.5% of physicians were labeled as high-cost outliers (100%-86.5%=13.5%).

Note, because of ties in physician cost profile scores the percentages don’t exactly match up (17.7 vs. 17.8%). We have also added two statistical testing columns:

1. Rank-ordered the t-tests and looked at all the t-tests with associated p-value >0.05, but still in the lowest 25% in terms of costs.
2. Rank-ordered the t-tests and identified all the t-test values with an associated p-value of >0.05 but still in the highest 25% in terms of costs.

With this 5x5 table we can again look at the “off-diagonal” cells as a measure of level of disagreement. In the 5x5 table for internal medicine the disagreement rate is 32.8%. In Table A.4 below we compare the disagreement rate shown in the manuscript to the disagreement rate using the 5x5 table described above. Across the specialties the disagreement rate does differ and sometimes a great deal. But the overall point of the manuscript remains, statistical testing will result in a significant fraction of physicians being placed in a different cost category.

We make two final points. The first is that we considered using the 5x5 table as our primary analysis for the manuscript. We chose against this analysis because it is not intuitive and quite complicated to explain. Also the comparison we made in the manuscript more likely reflects how the two techniques would be used in practice and the overall story does not change – statistical testing results in a significant fraction of physicians being categorized differently.

The second point is that the 5x5 tables illustrates another method of categorization which blends statistical testing and percentile cut-offs. Just as in statistical testing we created a t-test value for each physician which incorporates the uncertainty of the cost profile. But then similar to percentile cut-offs, one can rank-order the t-test values for each physician to identify the top and bottom 25% of physicians. This blending technique is similar to what was used by a co-author in a previous manuscript.(5) In that manuscript, Thomas and colleagues use what they term the standardized cost difference (SCD) which mimics a t-test value (one difference is that standard errors are calculated in a different manner in this manuscript). They then rank-ordered the physicians based on their SCDs and identified the top 10%.

**Table A.4 – Comparison of Disagreement Rate Using 3x3 table and 5x5 tables**

|  | Disagreement reported in manuscript with 3x3 table | Disagreement with 5x5 table |
| --- | --- | --- |
| Allergy and Immunology* | 27% | - |
| Cardiology | 34% | 25% |
| Cardiothoracic Surgery | 44% | 22% |
| Dermatology* | 22% | - |
| Emergency Medicine | 36% | 17% |
| Endocrinology | 35% | 33% |
| Family/General Practice | 30% | 35% |
| Gastroenterology | 19% | 25% |
| General Surgery | 41% | 24% |
| Hematology/Oncology | 38% | 32% |
| Infectious Diseases | 33% | 39% |
| Internal Medicine | 27% | 33% |
| Nephrology | 43% | 33% |
| Neurological Surgery | 32% | 16% |
| Neurology | 26% | 23% |
| Obstetrics and Gynecology | 38% | 26% |
| Ophthalmology | 20% | 24% |
| Orthopedic Surgery | 33% | 17% |
| Otolaryngology | 23% | 27% |
| Physical Medicine & Rehabilitation | 29% | 23% |
| Plastic Surgery | 29% | 24% |
| Psychiatry | 29% | 21% |
| Pulmonary & Critical Care | 41% | 26% |
| Rheumatology | 32% | 28% |
| Urology | 34% | 26% |
| Vascular Surgery | 46% | 14% |

* 5x5 table cannot be created for these two specialties because they have greater than 25% outliers using statistical testing in one or both tails.

**References:**
